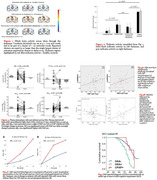# Assessing the impact of adequate OSA treatment on markers of sleepiness related to cognition and AD pathology among *Black* and *Hispanic* subjects

**DOI:** 10.1002/alz.095587

**Published:** 2025-01-09

**Authors:** Omonigho M Bubu

**Affiliations:** ^1^ NYU Grossman School of Medicine, New York, NY USA

## Abstract

**Background:**

Obstructive Sleep Apnea (OSA) and Excessive Daytime Sleepiness (EDS) are associated with increased Alzheimer’s disease (AD) risk. Black and Hispanic subjects have a higher burden of AD, present with greater OSA symptom severity, and EDS than non‐Hispanic whites. We present preliminary data supporting an innovative trial examining the impact of a novel OSA treatment paradigm on markers of (i) sleepiness related to cognition and (ii) AD progression, among *Black* and *Hispanic* subjects.

**Method:**

Our proposal offers a novel wholistic “personalized multi‐modal (PMM) OSA treatment paradigm that addresses socio‐structural determinants of health (sSDOH) that pose individual and system‐level barriers to OSA treatment adherence. We assess the degree of effective AHI and sleepiness reduction (AHI3A<15 and ESS<11) on the degree of short (3‐mo), intermediate (12‐mo), and long‐term (24‐mo) improvements in: 1) brain markers of sleepiness related to cognition measured by thalamic brain activity in a fMRI‐sustained attention task (at 3 and 24 mo); 2) AD (amyloid (A), tau (T) and neurodegeneration (N)) biomarker levels measured both in the brain (at 24 mo) and plasma (at 3, 12, and 24 mo); and 3) measured global cognitive performance (at 12, and 24 mo.). 160 subjects will be randomized to either undergo PMM or standard OSA treatment with CPAP only (i.e., 80 [40 Black & 40 Hispanic] subjects in each arm).

**Result:**

Our preliminary data suggest adequate OSA treatment: 1) improves thalamic brain activity in a fMRI‐sustained attention task with improved attention correlating with pupillometry metrics i.e., pupillary unrest index (PUI); 2) prevents OSA acutely increasing plasma NfL and tau and decreasing plasma Ab42/Ab40, and that OSA is associated with longitudinal brain amyloid and tau burden changes over 2 years; and 3) is associated with delayed onset of cognitive decline. *P< .05 for all*

**Conclusion:**

Adequate OSA treatment may impact AD progression in minoritized populations.